# Supplementary material for: Identification of shared bacterial strains in the vaginal microbiota of related and unrelated reproductive-age mothers and daughters using genome-resolved metagenomics
Source: PLoS One. 2022 Oct 26;17(10):e0275908. doi: 10.1371/journal.pone.0275908 (PMC9604009; doi:10.1371/journal.pone.0275908)
Supplement: Attachment — Submitted filename: mother_daughter_r2r.docx [file pone.0275908.s003.docx]

# Response to reviewer’s comments

Responses to comments in blue

Reviewer #1: This work aims to identify shared bacterial strains in vaginal microbiota of reproductive-age daughters and mothers (ages 14-27, 32-51; n=39 pairs) from a metropolitan area to evaluate the hypothesis that vertical transmission is present in vaginal microbiota. Overall similarity in microbiota composition was evaluated for all volunteers with metataxonomics, and additional genome-resolved metagenomics was used to detect shared strains in a subset of families (n=22). In general, metataxonomic similarities did not exceed random expectations and the strain-level metagenomic approach indicated that less strains were shared between family members (this correlated with age; younger daughters shared more strains with their mothers) than unrelated pairs, one explanation being that biogeography might be a stronger driver for similarities in the vaginal microbiota of reproductive-age women than family relations, although some evidence for vertical transmission was present.

I focus on bioinformatics aspects of the work in the evaluation.

Overall, the methodology is sound and clearly reported, and the conclusions are supported by the presented material. Standard methods for bioinformatics and data analysis have been used; it is interesting to note the use of the less commonly used Yue-Clayton similarity index in the analysis. Limitations of the study are discussed adequately, and relevant literature is cited.

I commend the authors for not only making the data and code available but doing this in a well-organized format including an open license (GPL-3).

I only have the following minor comments on this report.

We thank the reviewer for their time and effort in evaluating our work.

\# Minor

- Fig 1: might be useful to check if there are alternative ways to visualize the M/D relations in this figure; the comparison is complicated by the large number of colors (species) and limited overlap in M/D pairs. Perhaps showing the results per species & group level variations across all subjects (e.g. difference in the abundance between M/D?) could help to highlight the most
distinguished similarity patterns. Just a suggestion to consider.

We explored additional options for how best to display Figure 1 but ultimately chose to keep the current version. A motivating reason for our decision was that the current plot allows us to display the family’s community composition and label them by number. These numbers are re-used in the network diagram and allow the reader to refer back to the family’s community composition.

- Consider using a more permissive and broadly compatible open source
license, such as MIT, BSD-2-clause, or Artistic 2 (for discussion,
see e.g. Morin et al. 2012
[https://journals.plos.org/ploscompbiol/article?id=10.1371/journal.pcbi.1002598](https://nam11.safelinks.protection.outlook.com/?url=https%3A%2F%2Fjournals.plos.org%2Fploscompbiol%2Farticle%3Fid%3D10.1371%2Fjournal.pcbi.1002598&data=05%7C01%7CMFrance%40som.umaryland.edu%7Caabee404d115472f28ff08da91eb0603%7C717009a620de461a88940312a395cac9%7C0%7C0%7C637982735846208686%7CUnknown%7CTWFpbGZsb3d8eyJWIjoiMC4wLjAwMDAiLCJQIjoiV2luMzIiLCJBTiI6Ik1haWwiLCJXVCI6Mn0%3D%7C3000%7C%7C%7C&sdata=0PHVMXuTr5zwoUWoQtvp%2Be%2FrmJEWKIzKSKX%2BQhYuLFY%3D&reserved=0)). Also
consider that many open source developers (including Linux kernel)
have chosen to stick to GPL2.

We have revised the open-source license to the more permissive MIT, as suggested by the reviewer.

- A README file in the source code could help to navigate and
interpret the material

A README file was added to the Github page to help navigate the provided material.

- line 182; was the Student's t test assumptions fulfilled (approximately normally distributed groups), or would a non-parametric test (Wilcoxon) be justified?

Upon further examination, the assumption of normality was not met. For this reason, we have switched to the non-parametric test as suggested by the reviewer. Note the p-value did not change.

L174-176 “Sequence similarity between shared strains identified in the same family versus different families were compared using a Wilcoxon rank sum test.”

L242-244 “We found strains shared within families trended to being more similar to one another than those shared between families, but this difference was not significant (Figure 5, W_15.1_=-136, p=0.149).”
